# A Systematic Single-Parameter Study for the Melt Electrospinning of Poly(lactic acid)

**DOI:** 10.3390/polym18202551

**Published:** 2026-10-16

**Authors:** Rosa Barranco-García, Coro Echeverria, María L. Cerrada, Alexandra Muñoz-Bonilla, Marta Fernández-García

**Affiliations:** Instituto de Ciencia y Tecnología de Polímeros (ICTP-CSIC), C/Juan de la Cierva 3, 28006 Madrid, Spain; rbarranco@ictp.csic.es (R.B.-G.); cecheverria@ictp.csic.es (C.E.); mlcerrada@ictp.csic.es (M.L.C.); sbonilla@ictp.csic.es (A.M.-B.)

**Keywords:** melt electrospinning, poly(lactic acid), biodegradable scaffolds, process optimization, fiber morphology

## Abstract

Melt electrospinning (MES) is a fiber manufacturing technique in which a molten polymer is stretched into ultrafine fibers by an electric field. MES combines the ability to produce very fine fibers with the advantages of solvent-free manufacturing. Its controlled variant, melt electrowriting (MEW), is especially valuable because it allows exceptional architectural precision, making it highly attractive for biomedical applications. This study presents a systematic, single-parameter evaluation of how nozzle-to-collector distance (nozzle height), applied pressure, and collector speed influence the morphological and thermal properties of the resultant poly(lactic acid) (PLA) fibers. Fiber diameter, uniformity, thermal transitions, crystallinity, and thermal stability are analyzed, the former being tuned over a wide controlled range (≈4–42 μm). Larger nozzle heights and higher collector speeds promote fiber drawing, yielding thinner (down to ~4 µm) and more uniform fibers, while increasing pressure results in progressively thicker fibers due to higher melt flow rates (up to ~42 µm). Thinner fibers exhibit higher glass transition temperatures, suggesting enhanced molecular orientation, whereas all fibers remain amorphous due to the rapid quenching applied during processing. Thermal stability improves at intermediate nozzle heights, correlating with the increased morphological uniformity. These results provide a clear, quantitative understanding of the physical mechanisms governing MES processing of PLA, delivering practical guidelines and processing–structure–property relationships for the design of tailored biodegradable scaffolds.

## 1. Introduction

Fabrication of polymeric micro- and nanoscale fibers has become a central topic in materials science due to their unique combination of a high surface-to-volume ratio, tunable porosity, and mechanical adaptability [1]. These characteristics highlight their use in a broad range of fields, including catalysis, sensing, filtration, protective textiles, and—most prominently—biomedical engineering [2,3]. In tissue engineering, fibrous scaffolds are particularly valuable because they mimic the structural and functional features of the native extracellular matrix (ECM), providing an environment that promotes cell adhesion, proliferation, migration, and differentiation [4]. Micro- and nanofibers can reproduce the length, scale, and organization of ECM fibrillar components, enabling the design of biologically responsive architectures that support tissue regeneration [5,6].

Among the various fiber-fabrication techniques, electrospinning is one of the most established and versatile. It enables production of continuous fibers from the micrometer range down to several nanometers, and it has been widely applied to generate non-woven mats with ECM-like morphology. Such fiber networks, with their high surface area and tunable porosity, are effective in enhancing protein adsorption and promoting cell–material interactions essential for tissue repair [7]. However, despite its versatility, conventional electrospinning relies heavily on organic solvents. Residual solvent traces can compromise biocompatibility, induce cytotoxicity risks, complicate regulatory approval, and increase processing costs due to the required solvent handling, its removal, and subsequent recycling steps [8,9,10].

To overcome these limitations, solvent-free alternatives, such as melt electrospinning (MES) and melt electrowriting (MEW), have gained significant attention. MEW, in particular, represents a major technological advancement: it merges the principles of melt electrospinning with computer-controlled additive manufacturing, enabling the precise deposition of microfibers in predefined architectures.

Unlike traditional electrospinning, which typically produces random non-woven meshes, MEW allows high spatial resolution and deterministic fiber placement, facilitating the fabrication of two- and three-dimensional structures with predefined patterns [11,12]. In MEW, fibers with thicknesses in the micrometer range are deposited from the melt with the aid of electrical voltage using a computer-controlled stage [13]. Important parameters of the MEW process are the temperature of the polymer melt, the pressure or feed rate, the speed of moving elements, such as the collector and/or the print head, and the nozzle height, defined as the distance between the nozzle and the collector. By tuning these parameters, the polymer melt can steadily be extruded and directly written onto a collector. Thus, different patterns can be shaped [4,7]. Besides this precision control over geometry, MEW is a non-contact printing technique and, therefore, is promising for manufacturing scaffolds with desirable geometries in a wide range of biomedical applications [14,15]. This level of precision has positioned MEW as one of the most promising platforms for producing customized porous scaffolds for biomedical applications, including tissue engineering, regenerative medicine, and the development of patient-specific medical devices [16,17]. Tubular architectures are critical components in tissue engineering, replicating both the morphology and the dynamic functions of anatomical conduits responsible for fluid flow, nutrient exchange, and physiological regulation [18,19,20]. Most studies rely on poly(caprolactone) (PCL), whose flexibility and excellent melt-processability make it far more suitable for MEW than other polymers. Nevertheless, PCL shows insufficient mechanical performance for applications requiring structural support.

Poly(lactic acid) (PLA) is a particularly attractive polymer for MES and MEW because it is biocompatible, biodegradable, derived from renewable resources, and widely accepted in biomedical applications. There are several molecular factors affecting PLA’s biodegradation behavior, including molecular weight, structure, stereochemistry, catalysts, temperature, pH, and the degree of crystallinity. The biodegradation of PLA takes place through two consecutive stages: (1) hydrolytic degradation of polymeric chains and (2) microbial assimilation and mineralization. The rate of PLA biodegradation depends on the environment conditions used. For instance, industrial composting (55–60 °C, high humidity, active microbes) leads to typical PLA disintegration and biodegradation within 45–180 days (about 1.5–6 months). In contrast, home composting at ambient conditions promotes a much slower biodegradation, which may take 1–2 years or longer, with incomplete degradation often observed. Furthermore, PLA is very slow to degrade in soil and natural environments, which can persist for more than 2 years under typical environmental conditions.

This ability to degrade into non-toxic by-products [9] and its well-established processing window make it a preferred material for temporary scaffolds designed to support tissue regeneration. However, the transition of PLA pellets into functional microfibers with controlled morphology and properties is strongly governed by a complex interplay of MES processing conditions. Key parameters such as melt temperature, applied pressure (which determines flow rate), collector speed (associated with mechanical drawing), and nozzle height (which affects electrostatic stretching and cooling) play crucial roles in determining fiber diameter, surface uniformity, crystallinity, mechanical behavior, and thermal performance [13]. Among these properties, fiber diameter is of particular relevance. It dictates essential scaffold features such as pore size, mechanical performance, and surface area and also influences key biological processes including protein adsorption, cell spreading, and cell infiltration [8,21]. Fine control over fiber diameter is, therefore, indispensable for tailoring scaffold architecture to specific tissue engineering applications. In fact, reducing fiber diameters improves cell adhesion, protein adsorption, and surface area [8].

A conceptual description of the process is useful to understand how each processing parameter contributes to the fiber formation due to the complexity of the forces acting on the polymer jet during MES/MEW. Scheme 1 provides the schematic representation of the process using flat and drum collectors.

The nozzle height defines the available flight time in which the electric field can stretch the jet before solidification. The applied pressure controls the melt flow rate at the nozzle, establishing the initial jet diameter. Meanwhile, the collector speed imposes a mechanical drawing force that further attenuates the jet during deposition. These combined effects operate within the solidification window and ultimately determine the final fiber diameter and uniformity. This conceptual framework establishes the physical basis for the systematic one-parameter investigation presented below and clarifies the mechanistic rationale behind the processing–structure–property relationships explored in this work. Although previous studies [22,23] have examined some of these MEW parameters individually, there is still a lack of systematic analyses that isolate the direct effect of each key parameter, correlating it simultaneously with the subsequent morphological and thermal properties of PLA fibers. Addressing this gap is essential for building a solid mechanistic understanding of the MEW process and for guiding future optimization studies, including design-of-experiments (DoE) approaches. In this work, we present a comprehensive, one-parameter-at-a-time investigation of the influence of nozzle height, applied pressure, and collector speed on the PLA fibers produced using a commercial MES system. The effects of these parameters on fiber diameter, homogeneity, crystallinity, and thermal stability are systematically evaluated through scanning electronic microscopy (SEM), differential scanning calorimetry (DSC), and thermogravimetric analysis (TGA). The goal is to establish robust processing–structure–property relationships, enabling identification of the most influential parameters that govern fiber formation. The insights gained from this study provide a foundation for the rational design of MES and MEW processes and contribute practical guidelines for tailoring the morphology and thermal properties of PLA fibers for applications in biomedicine, sustainable packaging, and technical textiles.

## 2. Materials and Methods

### 2.1. Materials

Poly(lactic acid) (PLA) (grade 3051D) was supplied by NatureWorks LLC, Blair, NE, USA. This PLA has a weight-average molecular weight (M_w_) and molecular weight distribution (M_w_/M_n_), determined from gel permeation chromatography (GPC), of 148.6 kg/mol and 1.94, respectively, a density of 1.25 g/cm^3^, a melt flow index of 6.5 g/10 min (190 °C, 2.16 kg), and a content in *D*-isomer units of about 4 mol% [24].

### 2.2. Melt Electrospinning of PLA Fibers

Fibers were fabricated using a NovaSpider apparatus (CIC nanoGUNE, San Sebastián, Spain). The system consists of a temperature-controlled printhead with air pressure-assisted dispensing and a drum collector of 70 mm in diameter. In the present work, the XY axes were maintained constant. A negative voltage of 10 kV was applied to the collector, and the nozzle with an internal diameter of 0.3 mm was grounded. The nozzle height (Z), the pressure (P), and the collector speed (S) were varied from 90 to 220 mm, from 5 to 200 kPa, and from 700 to 2000 rpm, respectively. During the process, the dispenser was kept at 260 °C while the nozzle was 270 °C. The electrowriting process was carried out at room temperature with a relative humidity of 45%. The fibers were generated using G-code commands. The resulting samples were named ZxPySv, i.e., Z90P5S1000 corresponds to the sample made at a height of 90 mm with a pressure of 5 kPa and at a collector speed of 1000 rpm.

### 2.3. Characterization Techniques

Fibers were evaluated by SEM using a Philips XL30 microscope (Philips, Eindhoven, The Netherlands) with an acceleration voltage of 25 kV. The samples were coated with a layer of 80:20 Au/Pd alloy and deposited in a holder before visualization. To calculate the fiber diameter after taking the images, ImageJ v1.53k software was used.

DSC was used to determine the thermal transitions of fibers and was carried out in a TA Instruments (New Castle, DE, USA) Q100 calorimeter connected to a cooling system and calibrated with different standards. The sample weights were around 3 mg. A temperature interval from 20 to 180 °C was studied at a heating rate of 10 °C/min. A value of 93.6 J/g was used as the enthalpy of fusion of a perfectly crystalline PLA material [7,10], although values are reported to fluctuate between 80 and 135 J/g, depending upon the calculation method used [25]. Thermogravimetric analysis (TGA) was performed using Q500 equipment by TA Instruments (New Castle, DE, USA) under a nitrogen atmosphere at a heating rate of 10 °C/min.

### 2.4. Statistical Analysis

Analysis of variance (ANOVA) was performed to detect any significant differences between the factors at *p* < 0.05. The software used was Origin 8.5 (OriginLab Corporation, Northampton, MA, USA).

## 3. Results

The experimental approach in this study is designed as a systematic, one-variable-at-a-time investigation. While a full factorial design-of-experiments (DoE) could model complex interactions, it was considered beyond the scope of this initial foundational work. The primary objective here is first to establish and clearly elucidate the individual, direct effects of each key parameter (nozzle height, pressure, and collector speed) on the fiber properties, particularly on the morphological and thermal properties. This initial knowledge is critical for the resultant fibers as it provides a clear physical understanding of the process and identifies the most influential parameters. The trends and relationships identified in this study will guide future DoEs by determining critical factors and their effective ranges [26,27]. It should also be noted that a reduction in the molecular weight of the PLA was noticed after fiber processing, this drop being independent of the parameter analyzed.

### 3.1. Collector Speed Effect

Different collector speeds were chosen in order to analyze its effect on the obtained fiber diameter. The collector speed governs the rate at which the fiber is drawn and collected, acting as a mechanical drafting force that has a direct effect on the fiber diameter, as is revealed below.

***Morphological Analysis***

Figure 1 and Figure 2 illustrate the effect of varying the collector speed from 700 to 2000 rpm at a constant nozzle height (90 mm) and pressure (5 kPa). As a general trend, a decrease in fiber diameter is observed with increasing speed, from 17.11 ± 1.88 µm at 700 rpm to 10.88 ± 2.86 µm at 2000 rpm.

Higher collector speeds involve a greater tensile force on the extruded polymer jet, leading to larger stretching and resulting in thinner fibers [13]. The reduction is not linear, highlighting the complex interplay existing between all parameters. The diameter is the highest at 700 rpm because the mechanical drawing may not have been sufficient to fully attenuate the specific flow rate, potentially leading to minor jet buckling or instability that transitorily increased the diameter [23,28].

***Thermal and Structural Properties***

The DSC analysis (Figure 3, Table 1) reveals a gradual decrease in T_g_ with increasing collector speed, from 54.5 at 700 rpm to 51.5 °C at 2000 rpm. This is an unexpected result, since molecular orientation is usually favored at higher drawing speeds, involving, consequently, restricted motions within the amorphous chains and a higher T_g_ value. In the current case, fibers become, however, slightly thinner at the highest speeds and their solidification might be somewhat faster, triggering formation of a larger number of micro-inhomogeneities and internal stresses that might lead to an increase in free volume. Consequently, this promotes segmental mobility within fibers, which implies a shift in T_g_ to lower temperatures. In these fibers, regardless of the collector speed, glass transition is fundamental as they are completely amorphous after processing, since enthalpies involved in the two thermal transitions (cold crystallization and melting event, commented below) above T_g_ lead to a zero neat total enthalpy of melting and, consequently, the total degree of crystallinity (X_c_^TOT^) during this first heating scanning is zero, as deduced from data listed in Table 1 [29,30].

A structural recovery process [31] is overlapped to glass transition, this superposition being a common occurrence in PLA [32,33]. Above T_g_, two additional thermal transitions (cold crystallization and the melting process) take place. Location of the former ranges from 109 to 117 °C, ordering being most favorable and appearing at inferior temperatures in the fibers achieved at the highest collector speeds. Crystals developed through cold crystallization from the glassy state are α-type, as the exothermic event is located around 109 °C. This assumption is based on (a) full absence of observation, to any extent, of transformation from α′ to α crystallites, and (b) the formation of only α′ crystals is described in the literature [34] to occur at cold crystallization temperatures below 80 °C for the PLA with this content in *D*-isomer. In the interval ranging from 80 to 90 °C, coexistence of α’ and α crystals is observed while the α polymorph is the only lattice attained above 90 °C.

The melting process begins once cold crystallization is over, appearing to be divided into two peaks, with their locations specified in Table 1. The smaller-temperature endotherm, at about 140 °C, most likely refers to the melting of the α crystals developed during cold crystallization, while the higher-temperature peak, at around 146 °C, is associated with the melting of recrystallized entities after their thickening on melting [35].

**Thermal stability**

The TGA results (Table 2, Figure 4) show some variation in the T_5%_ value with collector speed, but without a consistent trend. The values ranged from 254.5 °C to 286.5 °C, indicating that although speed influences fiber diameter and T_g_, its effect on the fundamental thermal degradation mechanism of PLA under these conditions is unclear. Similar behavior has been reported for PLA and PLA-based fibers produced by melt electrospinning or fused-filament methods, where T_5%_ and T_MAX_ values remain within a relatively narrow window and are dictated mainly by polymer chemistry and overall thermal history, not by moderate changes in drawing conditions [30,36].

### 3.2. Effect of Nozzle Height

The distance between the nozzle and the collector, here named as nozzle height, is a critical parameter that governs the flight time of the polymer jet, influencing the extent of fiber stretching, solvent evaporation (in solvent-based systems), and solidification. In melt electrospinning of PLA and PLA/PEG, a suitable nozzle height is required to allow sufficient whipping/stretching before solidification; heights around 10 cm were found to be optimal for obtaining uniform fibers, whereas heights that were too short led to rapid solidification and thicker, less uniform fibers [37,38]. In our solvent-free process, the primary effects are related to electrostatic stretching and cooling. The smallest fiber diameter using different collector speeds was attained at 2000 rpm. Accordingly, the value of 2000 rpm was selected as a constant parameter for manufacturing this new set of fibers.

***Morphological Analysis***

Figure 5 shows the SEM micrographs and corresponding diameter distributions of fibers produced at different nozzle heights (from 90 to 220 mm), while maintaining constant pressure (5 kPa) and collector speed (2000 rpm). A clear morphological observation is noticed: as the nozzle height increases, the fibers become progressively thinner and more uniform. The quantitative analysis in Figure 6 confirms this, showing a significant decrease in mean fiber diameter from 9.15 ± 2.81 µm at 90 mm to a minimum of 4.35 ± 0.53 µm at 200 mm. This reduction is attributed to the longer jet flight time, allowing enhanced electrostatic drawing before the polymer solidifies upon contact with the collector. Similar reductions in fiber diameter with increasing nozzle height have been reported for PLA melt electrospinning and for others achieved by MEW, where greater flight time enables additional electrostatic stretching until cooling/solidification becomes limiting [26,37,38]. In addition to this, the diameter dispersion also decreased substantially (standard deviation decreased from 2.81 µm to 0.51 µm), confirming improved jet stability and, therefore, improved fiber homogeneity. Beyond this point, the electric field strength may weaken, or the polymer may solidify prematurely, reducing the effective drawing force, in line with the existence of optimal working heights reported for MES and MEW systems [26,38,39].

***Thermal and Structural Properties***

The DSC thermograms (Figure 7) and data in Table 3 reveal the thermal behavior of these fibers. The glass transition temperature (T_g_) shows a general growing trend with nozzle height, going from approximately 50 °C at 90 mm to 61 °C at 200 mm. This increase suggests that the produced fibers undergo greater stretching as larger heights are used, inducing a higher degree of molecular orientation and alignment of the amorphous chains. Thus, the overall chain mobility is restricted and a displacement in T_g_ to higher temperatures is observed [29,37].

Above glass transition, all fibers exhibit a cold crystallization exotherm, characterized by its T_c_^cold^, and a subsequent melting endotherm, with its characteristic T_m_. A zero neat total enthalpy of melting is estimated from the enthalpies involved in both thermal transitions, meaning a complete amorphous PLA character of these different fibers (see Table 2). This is a common feature for melt-electrospun PLA fibers, since rapid solidification produces amorphous PLA fibers that undergo cold crystallization upon heating, with crystallinity increasing only upon annealing [37]. Fast cooling and further solidification within the quenched polymer fibers do not provide enough time for crystal growth [29,38]. Furthermore, it appears that the location of cold crystallization is dependent on nozzle height, so that as height increases, cold crystallization occurs at lower temperatures. This fact could be related to the higher alignment of chains found in fibers produced at higher heights, which triggers three-dimensional ordering. In addition to displacement of its location, the width of cold crystallization is broadened, meaning formation of more heterogeneous crystals, which are again α-type because of, on one hand, the position of cold crystallization and, on the other hand, the absence of transition from α′ to α crystallites.

The melting process consists of a bimodal endotherm, where the main melting temperature, T_m_, appears at about 150 °C. As mentioned previously, the peak at the lowest temperature is associated with the melting of the thinner α crystallites developed during cold crystallization, while the higher-temperature event is ascribed to the melting of recrystallized entities along with those thicker crystals also developed during cold crystallization. As deduced from Figure 7, the amount of more perfect crystallites increases from a certain nozzle height, and peaks at the highest temperatures.

***Thermal stability***

The thermal stability of the fibers was evaluated by TGA (Figure 8, Table 4). The temperature at 5% mass loss (T_5%_), a common indicator of stability, improved with increasing nozzle height, reaching a maximum of 304.0 °C at 170 mm. This enhancement can be linked to the formation of more uniform and oriented fibers with fewer structural defects, as seen in the SEM images.

More aligned chains within fibers, i.e., fibers with a more oriented morphology, can better resist thermal degradation, as the onset of chain scission may be delayed in a more ordered and, consequently, more compact and denser system.

### 3.3. Effect of Applied Pressure

Applied pressure directly controls the polymer flow rate, and is expected to have an impact on fiber diameter. This parameter is evaluated whilst maintaining a constant collector speed of 2000 rpm and a nozzle height of 200 mm.

***Morphological Analysis***

Figure 9 and Figure 10 demonstrate that increasing the pressure, while maintaining a constant height and collector speed, results in a substantial increase in fiber diameter from 4.36 ± 0.53 µm to 21.22 ± 1.49 µm. This is a direct consequence of the higher volume of polymer extruded per unit time. If the electrostatic and mechanical stretching forces remain relatively constant, this increased mass flow results in the deposition of a thicker fiber. This finding is in excellent agreement with the literature on MES [23,27,28,39,40].

***Thermal and Structural Properties***

The DSC results (Figure 11, Table 5) show that T_g_ decreases with increasing pressure, from 60.0 °C at 5 kPa to 55.0 °C at 200 kPa, meaning that higher pressures applied to fibers promote mobility within their amorphous chains. The diameter of these two fibers produced at the greatest pressures is the largest, meaning that a cooling gradient could take place from the surface to the core, which might allow partial chain relaxation in the core and, consequently, less aligned amorphous chains compared to fibers with lower diameters. The importance of increasing the pressure is also detected in the PLA cold crystallization process, above glass transition and its associated physical aging. As in the rest of the fibers evaluated, the polymorph developed along the cold crystallization is α-type, since there is no evidence of α′ to α transition and formation of an α′ lattice has been described [41] to be hindered in PLA with this relatively high *D*-isomer content. Variation in pressure leads to two main effects: location of the exothermic event and the enthalpy involved. Cold crystallization shifts to higher temperatures with increasing pressure, meaning that three-dimensional ordering of PLA chains is hampered with pressure. This hindrance is also related to the second observation, so the enthalpy involved is reduced.

The shape of the melting endotherm is also affected by pressure, as deduced from Figure 11. At the smallest pressure applied in Z200P5S2000, melting is basically a unimodal peak because crystals are developed at the lowest temperature and their distribution size seems not to show great differences. A clear bimodal endotherm is, however, noticed in Z200P25S2000, where the smaller-temperature endotherm most likely corresponds to the melting of the α crystals developed during cold crystallization, and that at high temperature is ascribed to the melting of recrystallized entities after their thickening upon melting. In Z200P100S2000, bimodality persists although the low-temperature endotherm becomes the main one since cold crystallization of α crystals occurs at higher temperature, diminishing the extent of recrystallized entities. This feature is even more evident in Z200P200S2000, with the peak at the lower temperature becoming the primary one.

***Thermal stability***

The TGA data (Figure 12, Table 6) indicate that pressure has a less systematic effect on thermal stability compared to nozzle height. The T_5%_ values fluctuated between 290.5 °C and 313.5 °C without a systematic trend, suggesting that pressure-induced changes in diameter and morphology have a secondary effect on the onset of thermal degradation, compared to the intrinsic thermal stability of the PLA polymer itself.

Similar onset degradation temperatures (≈280–320 °C) have been reported for melt-electrospun PLA and PLA composite fibers, where moderate variations in morphology or crystallinity cause only modest shifts in degradation onset [30,36]. Moreover, studies on thermally sensitive polymers, such as poly(lactic-*co*-glycolic acid) (PLGA) and polycaprolactone (PCL), show that processing-related degradation is strongly governed by residence time and temperature in the melt rather than by geometric parameters like flow rate within narrow processing windows, supporting the minor influence of pressure on T_5%_ observed here [42,43].

The results presented above reveal clear but distinct effects for each processing parameter. However, because nozzle height, applied pressure, and collector speed have been investigated independently, a broader integrative perspective is needed to compare their relative influence on fiber formation and thermal behavior. To facilitate this global interpretation, Figure 13 provides a consolidated parameter–effect map that links each processing variable with its dominant physical effect and the resulting morphological and thermal outcomes. As shown, increasing the nozzle height enhances the available flight time and promotes electrostatic stretching, producing thinner and more homogeneous fibers that exhibit higher T_g_ and improved thermal onset stability. Conversely, an increase in the applied pressure elevates the melt flow rate, resulting in thicker fibers with reduced chain orientation and slightly lower T_g_. Collector speed primarily imposes a mechanical drawing force that attenuates fiber diameter, although the presence of local non-monotonic deviations reflects transient jet instabilities intrinsic to the MES process.

It is important to mention that the fiber is produced under processing conditions that induce molecular stretching. This leads to an alignment of the polymer chains, which facilitates their ability to adopt ordered conformations upon heating. Consequently, cold crystallization can change its location as well as the enthalpy involved, as less energy is required for the chains to reorganize into crystalline domains. Results have shown that a rise in collector speed facilitates alignment as well as the nozzle height. However, rising pressure provokes a rapid cooling with lower oriented regions and, therefore, higher T_c_^cold^ values. Irrespective of the specific crystallization pathway of the fibers, the resulting total degree of crystallinity is essentially negligible, becoming fully amorphous.

Overall, this map offers an integrated view of the processing–structure–property relationships established throughout the study. This integrative visualization also highlights which parameters exert the strongest influence on PLA fiber morphology and thermal response, serving as a practical guide for selecting conditions tailored to specific scaffold requirements.

## 4. Conclusions

This systematic study successfully demonstrates the great and controllable influence of key melt electrospinning processing parameters on the properties of poly(lactic acid) fibers, establishing clear processing–structure–property relationships. We demonstrate that fiber diameter can be designed across a wide range (≈4 µm to > 40 µm) by controlling key parameters: increasing nozzle height (90 → 200 mm) for thinner, uniform fibers via enhanced electrostatic drawing; increasing applied pressure (5 → 200 kPa) for thicker fibers via higher flow rates; and modulating collector speed (700 → 2000 rpm) for mechanical drafting. The thermal properties are intrinsically linked to this designed morphology, with thinner fibers exhibiting higher glass transition temperatures due to molecular orientation, while all fibers produced at the different conditions are amorphous due to the rapid quenching applied. The clear relationships established here provide a foundational and essential step for the field, offering the physical insight required to guide future statistically powered optimization studies.

Finally, this work provides practical design guidelines for creating application-specific PLA scaffolds. For fine, cell-interactive structures in tissue engineering, a strategic combination of a high nozzle height, low pressure, and high collector speed is recommended. On the contrary, elevated applied pressures are suggested for fabricating robust structures where mechanical support or rapid deposition is paramount, as larger fiber diameters and higher line deposition rates have been exploited for mechanically reinforced or high-aspect-ratio architectures. This framework enables the precise engineering of advanced, customizable biodegradable scaffolds for regenerative medicine.

## Figures and Tables

**Figure 1:**
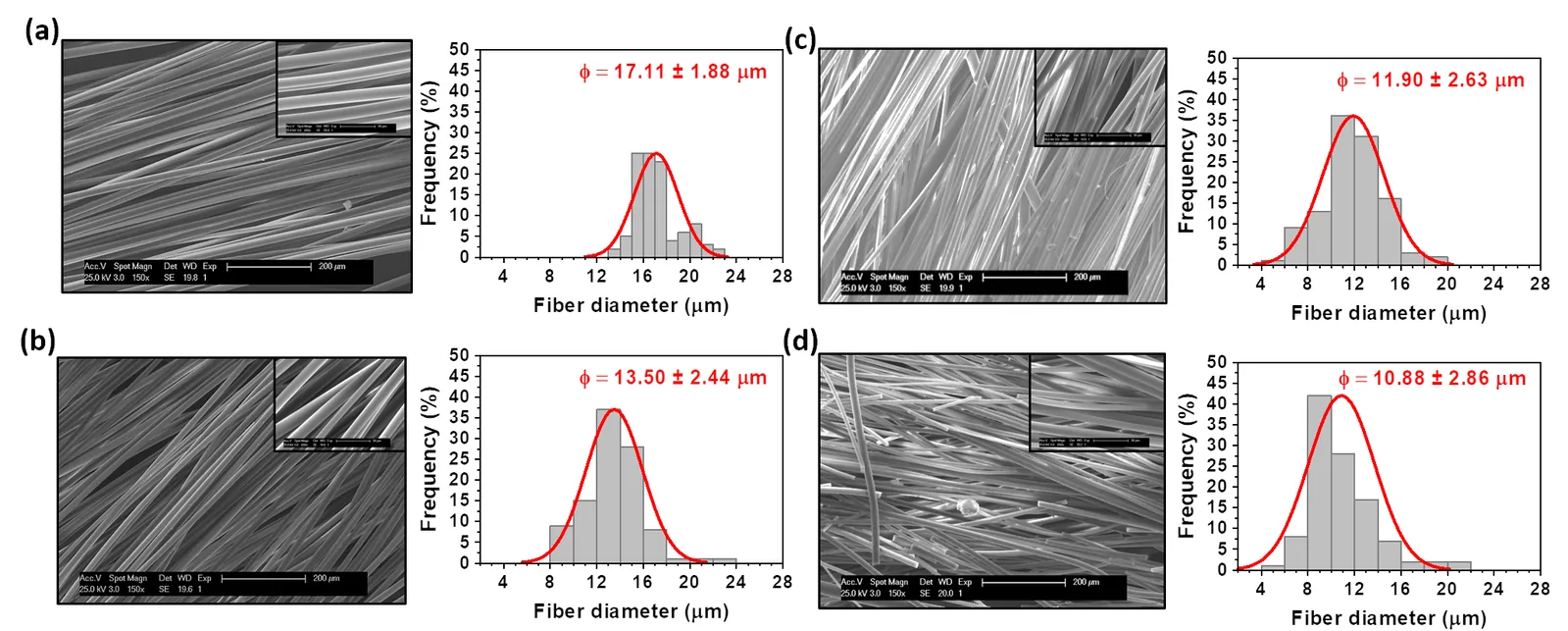
SEM images and fiber diameter variation in PLA fibers produced at collector speeds of (**a**) 700 rpm, Z90P5S700; (**b**) 1000 rpm, Z90P5S1000; (**c**) 1500 rpm, Z90P5S1500; and (**d**) 2000 rpm, Z90P5S2000 (nozzle height: 90 mm, constant pressure: 5 kPa).

**Figure 2:**
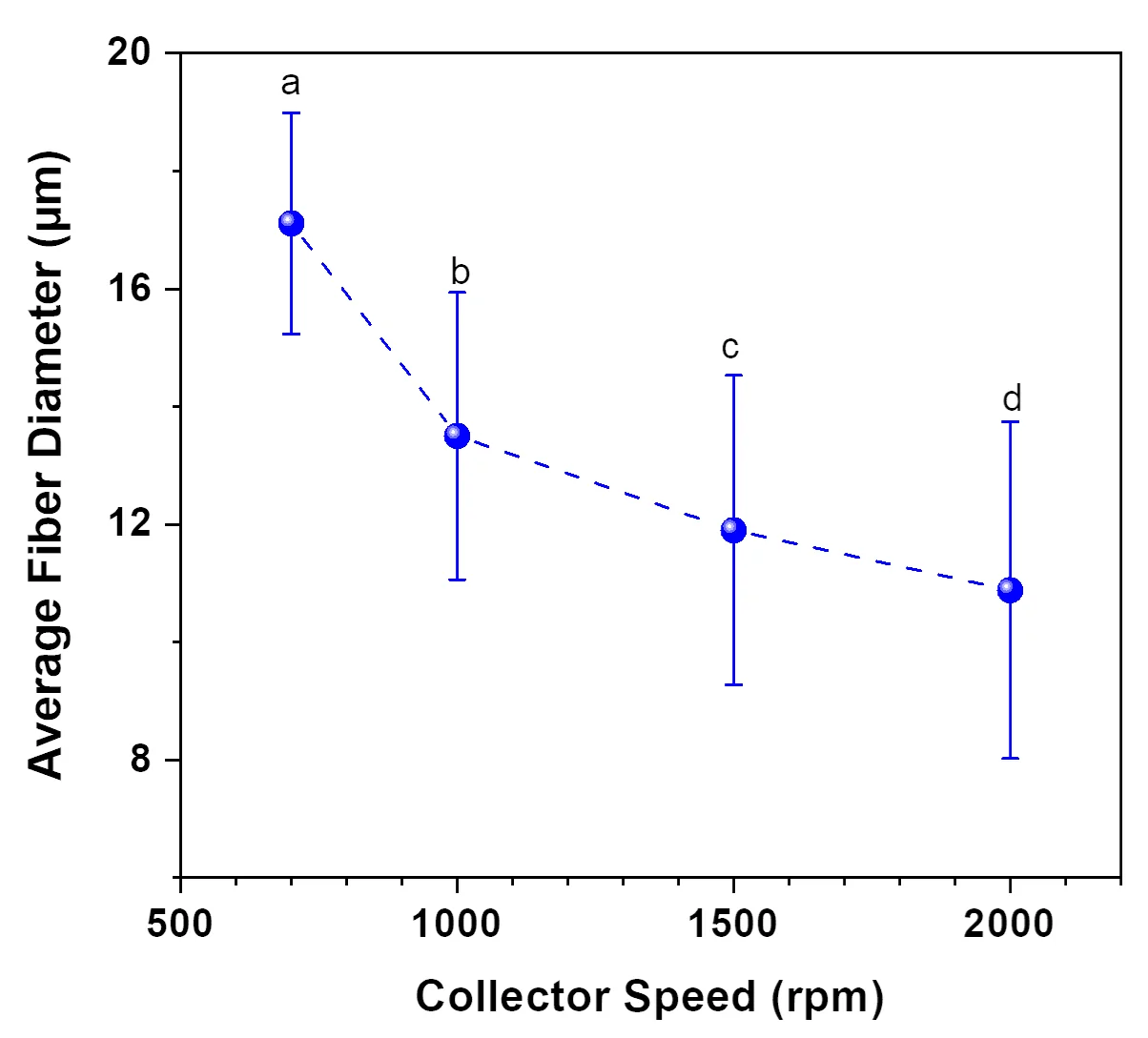
Fiber diameter variation with the collector speed (with a pressure of 5 kPa and constant nozzle height of 90 mm). Different letters mean significant differences between samples.

**Figure 3:**
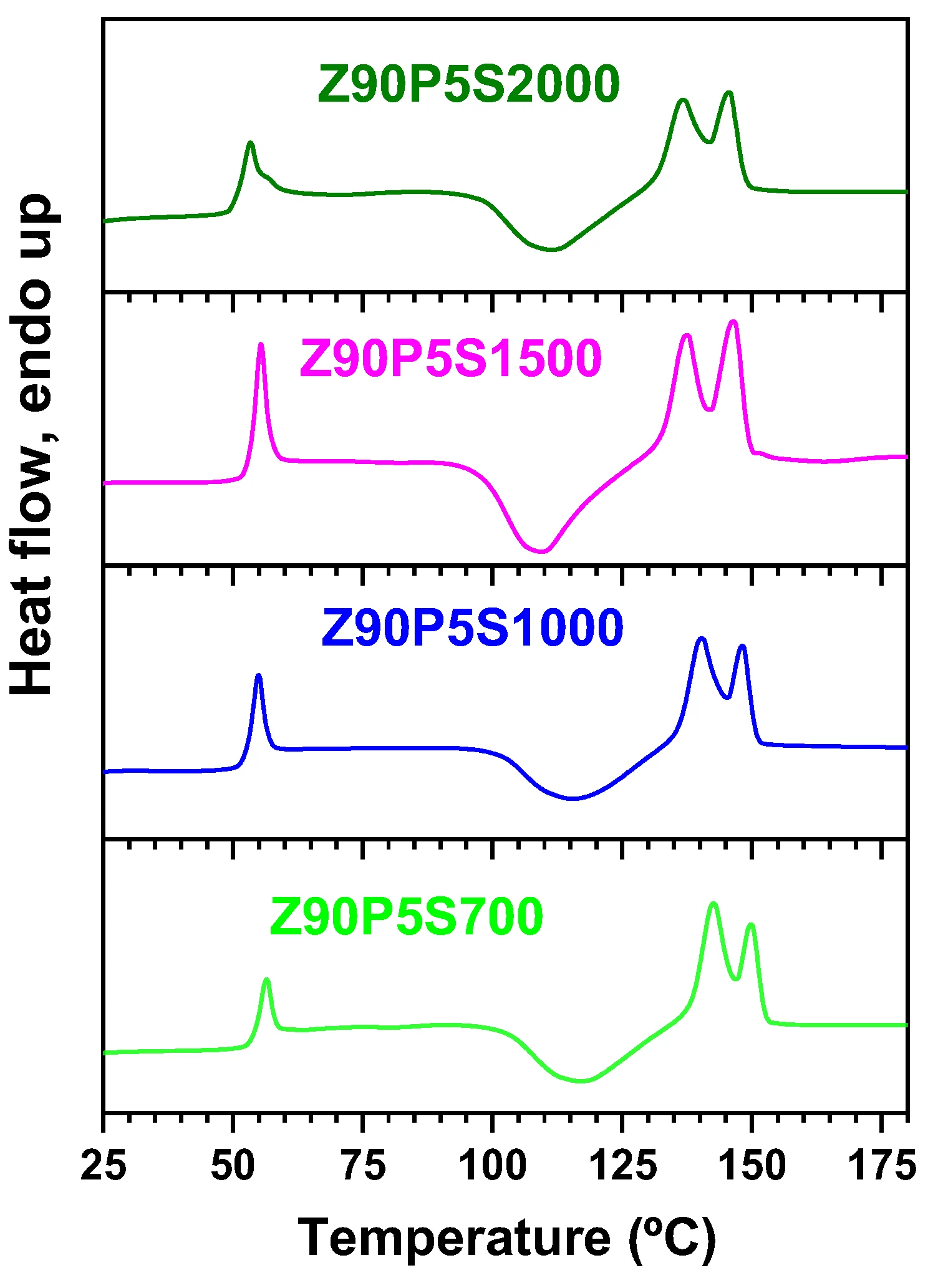
Thermograms corresponding to PLA fibers obtained at different collector speeds, a constant pressure of 5 kPa, and a constant nozzle height of 90 mm.

**Figure 4:**
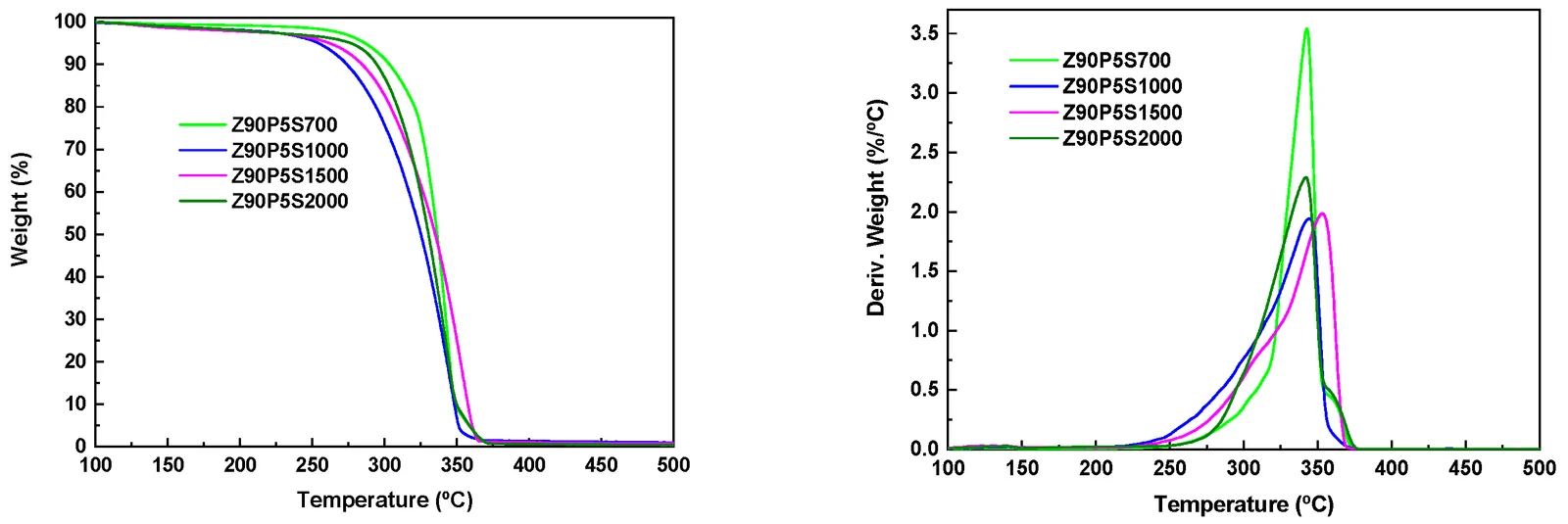
TGA results of PLA fibers produced at different collector speeds, a constant pressure of 5 kPa, and a constant nozzle height of 90 mm.

**Figure 5:**
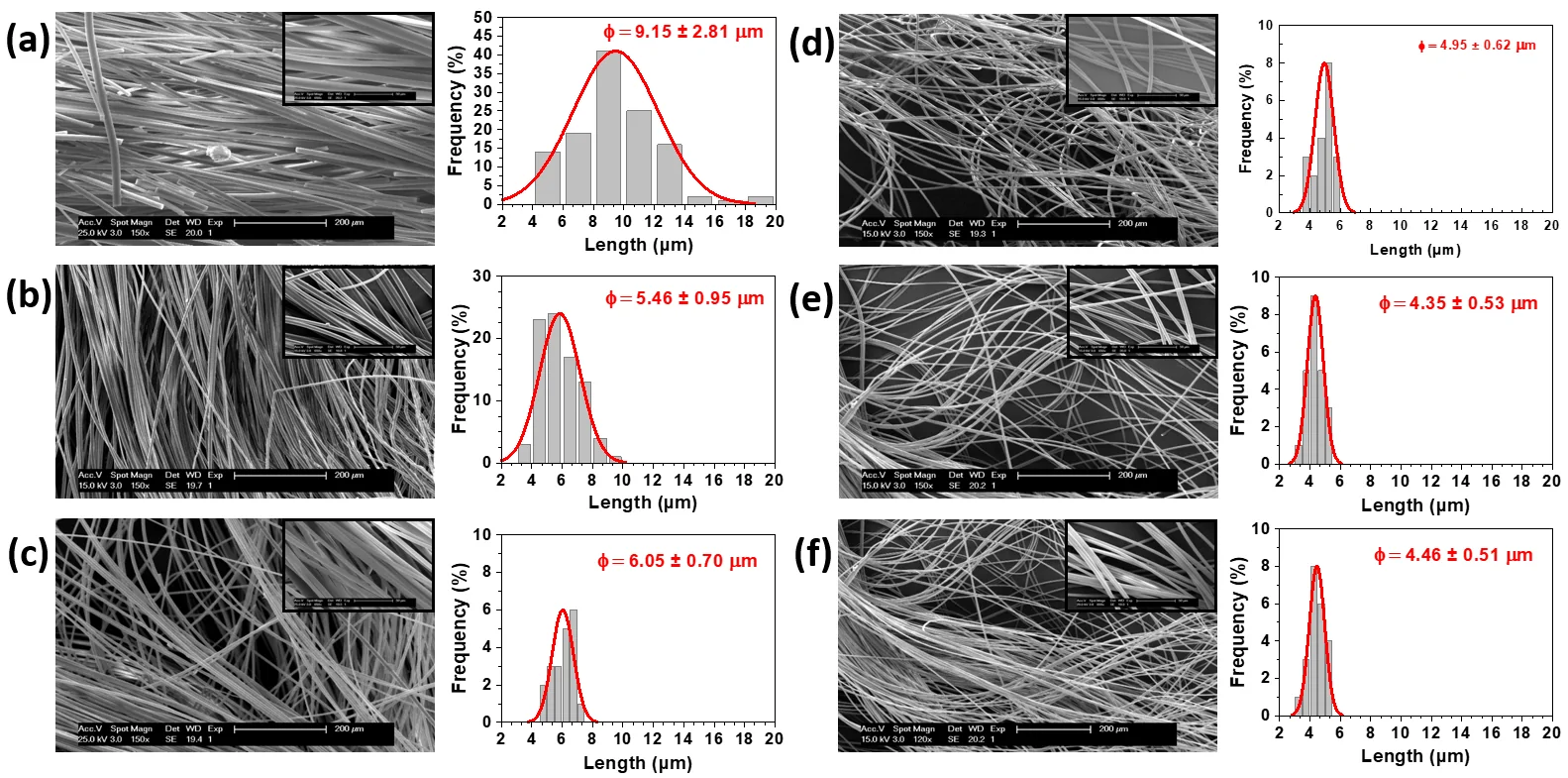
SEM images and fiber diameter variation in PLA fibers produced at nozzle heights of (**a**) 90 mm, Z90P5S2000; (**b**) 120 mm, Z120P5S2000; (**c**)150 mm, Z150P5S2000; (**d**) 170 mm, Z170P5S2000; (**e**) 200 mm, Z200P5S2000; and (**f**) 220 mm, Z220P5S2000 (constant pressure: 5 kPa, collector speed: 2000 rpm).

**Figure 6:**
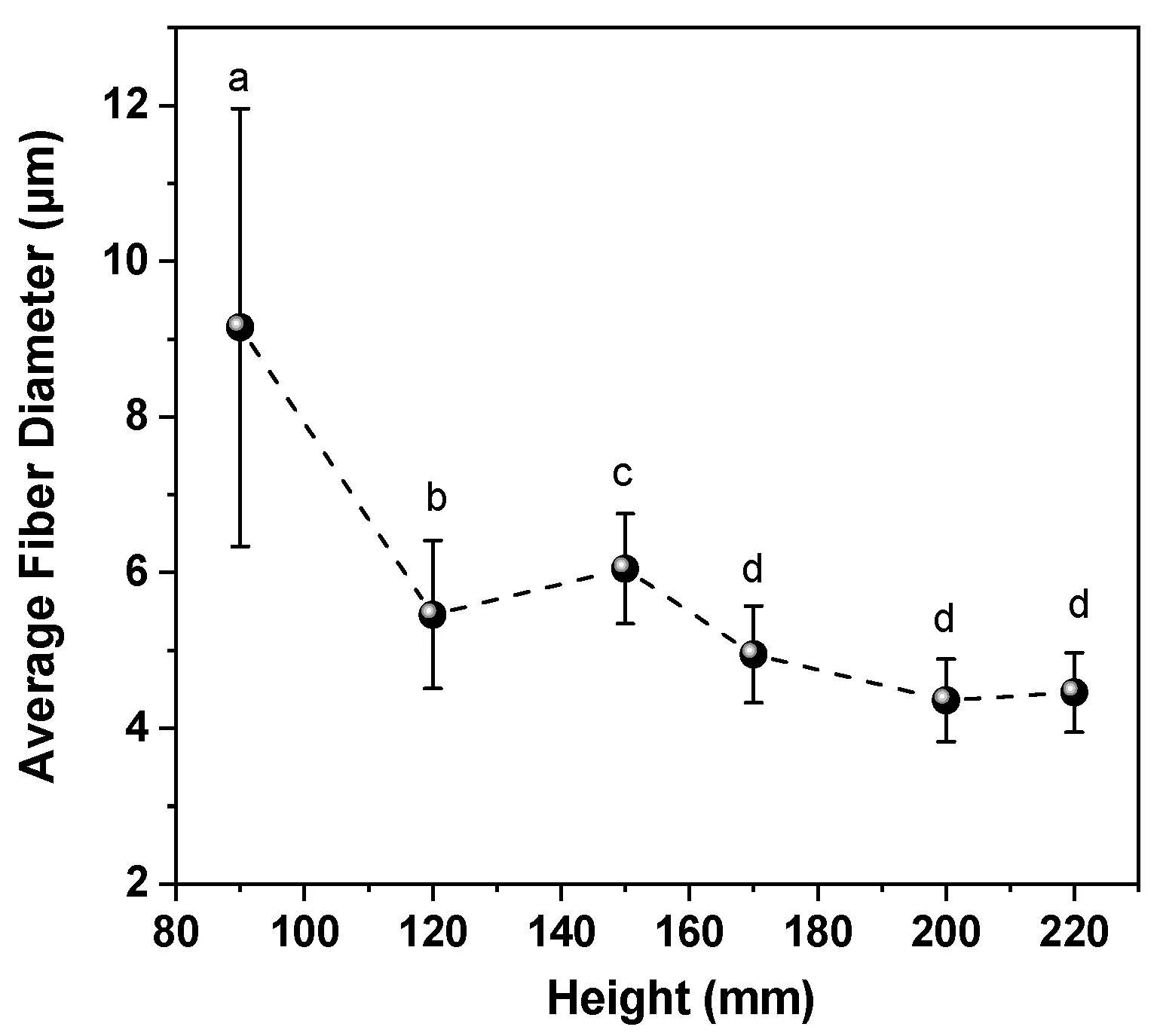
Fiber diameter variation with nozzle height at a pressure of 5 kPa and a collector speed of 2000 rpm. Different letters mean significant differences between samples.

**Figure 7:**
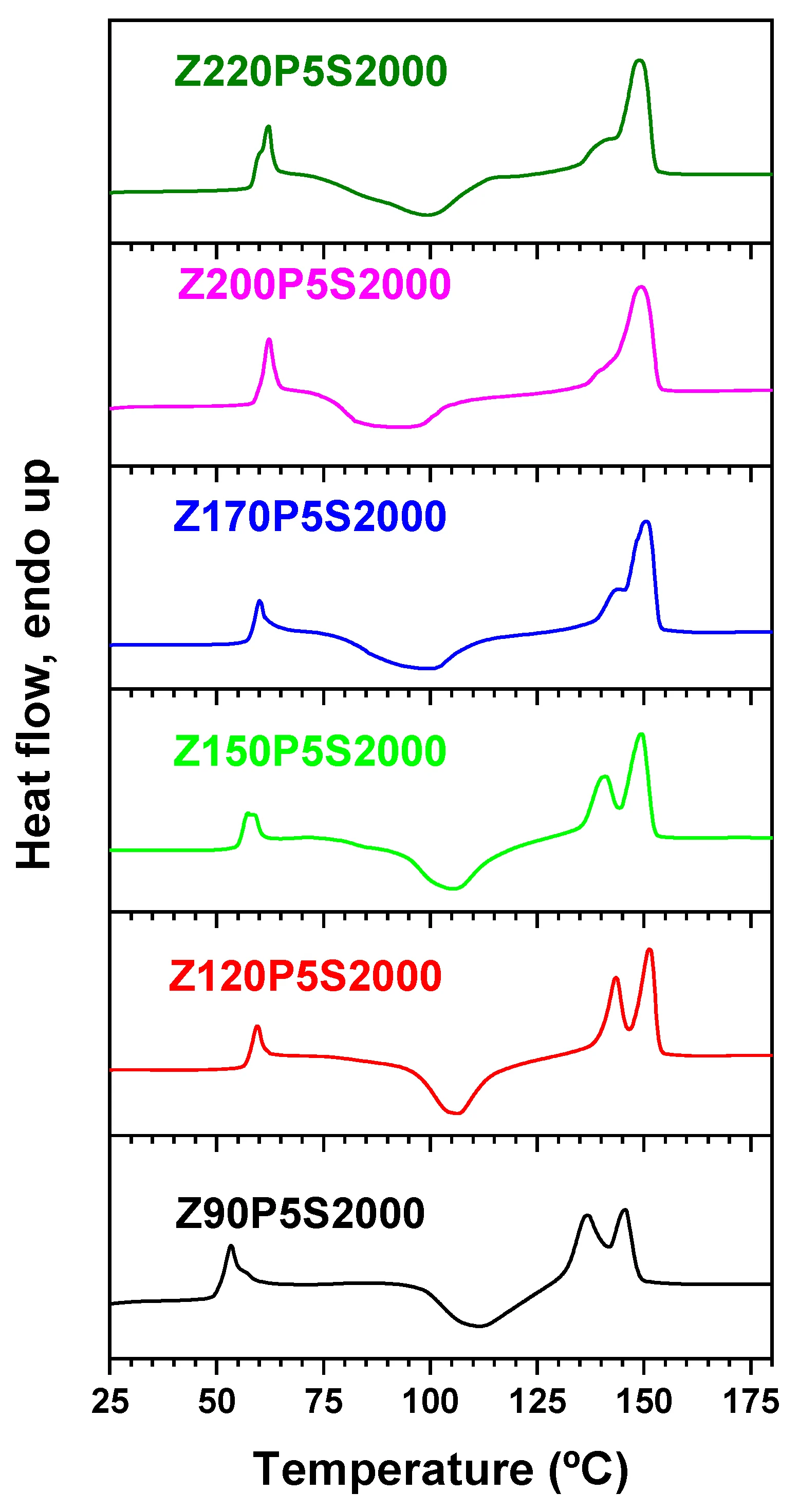
Thermograms corresponding to PLA fibers produced at different nozzle heights (constant pressure of 5 kPa, collector speed of 2000 rpm), from top to bottom: 220 mm, Z220P5S2000; 200 mm, Z200P5S2000; 170 mm, Z170P5S2000; 150 mm, Z150P5S2000; 120 mm, Z120P5S2000; 90 mm, Z90P5S2000.

**Figure 8:**
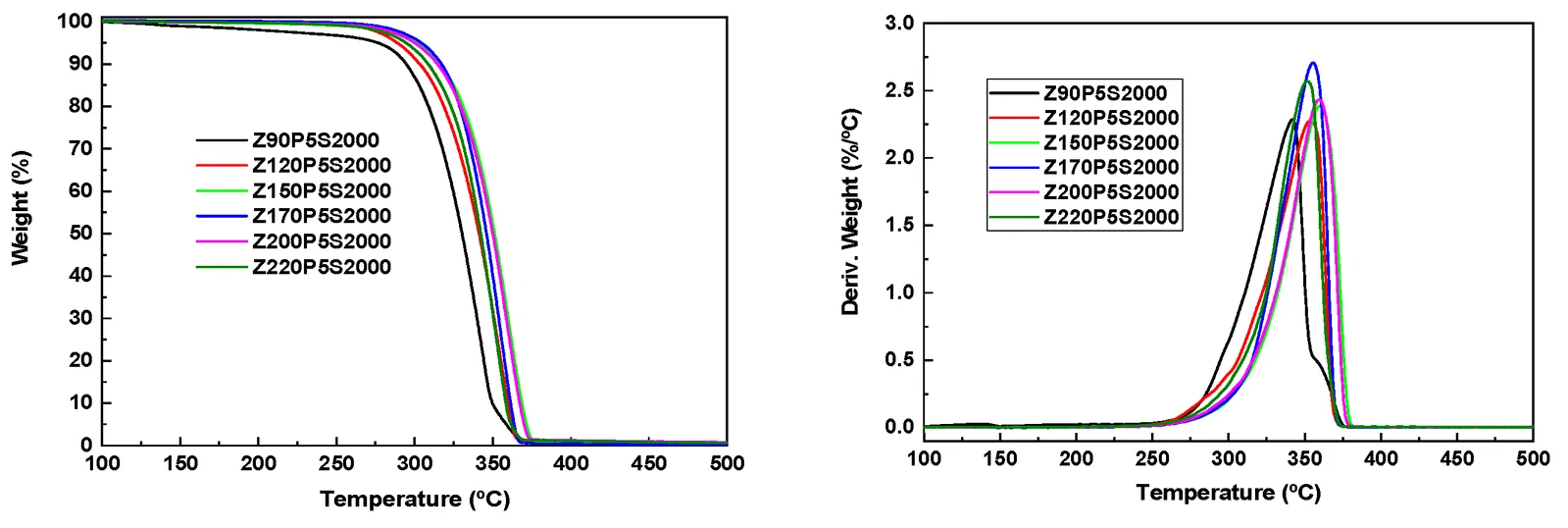
TGA curves of PLA fibers obtained at different nozzle heights: 90 mm, Z90P5S2000; 120 mm, Z120P5S2000; 150 mm, Z150P5S2000; 170 mm, Z170P5S2000; 200 mm, Z200P5S2000; and 220 mm, Z220P5S2000 (constant pressure of 5 kPa, collector speed of 2000 rpm).

**Figure 9:**
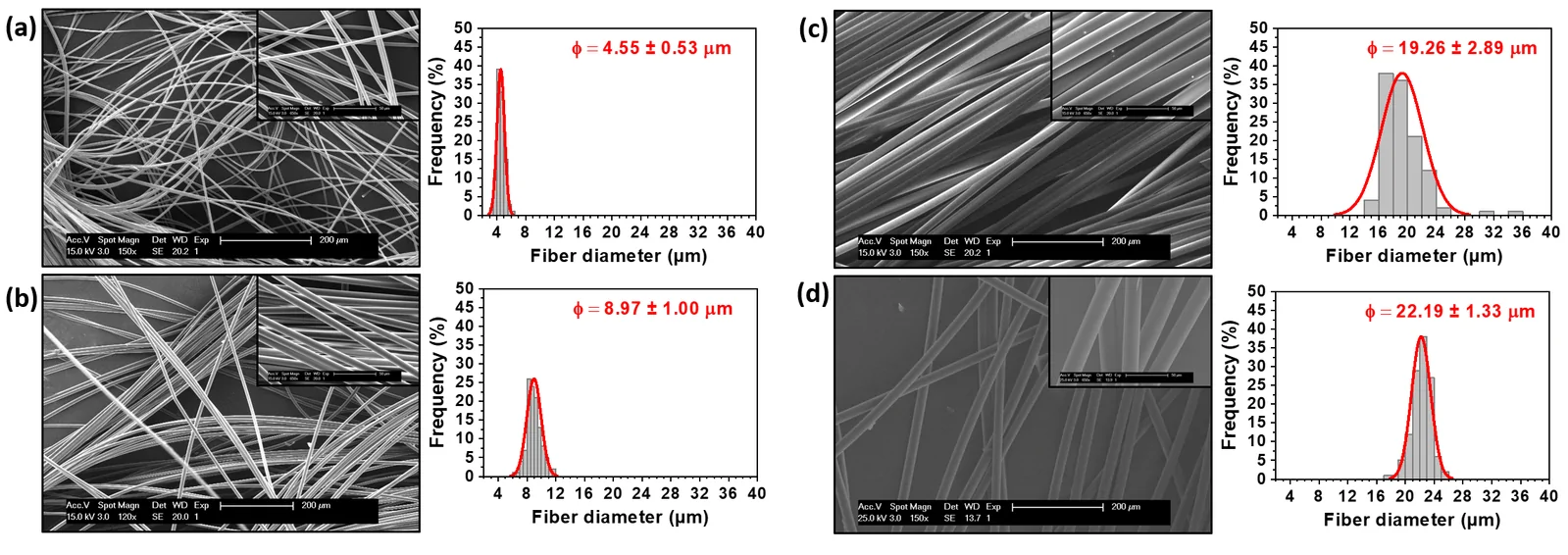
SEM images and fiber diameter variation in PLA fibers produced at the following pressures: (**a**) 5 kPa, Z200P5S2000; (**b**) 25 kPa, Z200P25S2000; (**c**) 100 kPa, Z200P100S2000; and (**d**) 200 kPa, Z200P200S2000 (nozzle heights of 200 mm, collector speed of 2000 rpm).

**Figure 10:**
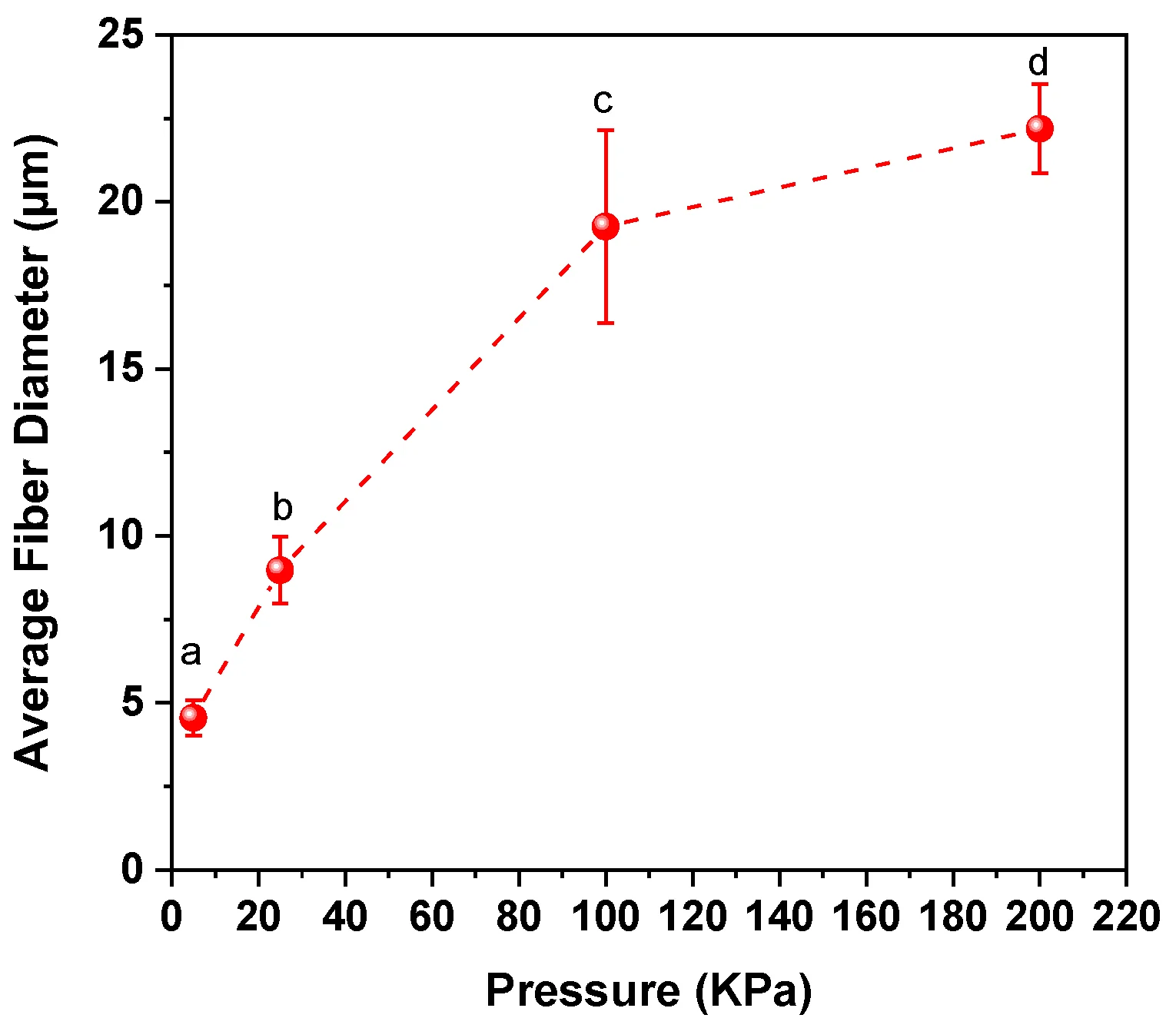
Fiber diameter variation with pressure when the height between the nozzle and the collector is 200 mm and the collector speed is 2000 rpm. Different letters mean significant differences between samples.

**Figure 11:**
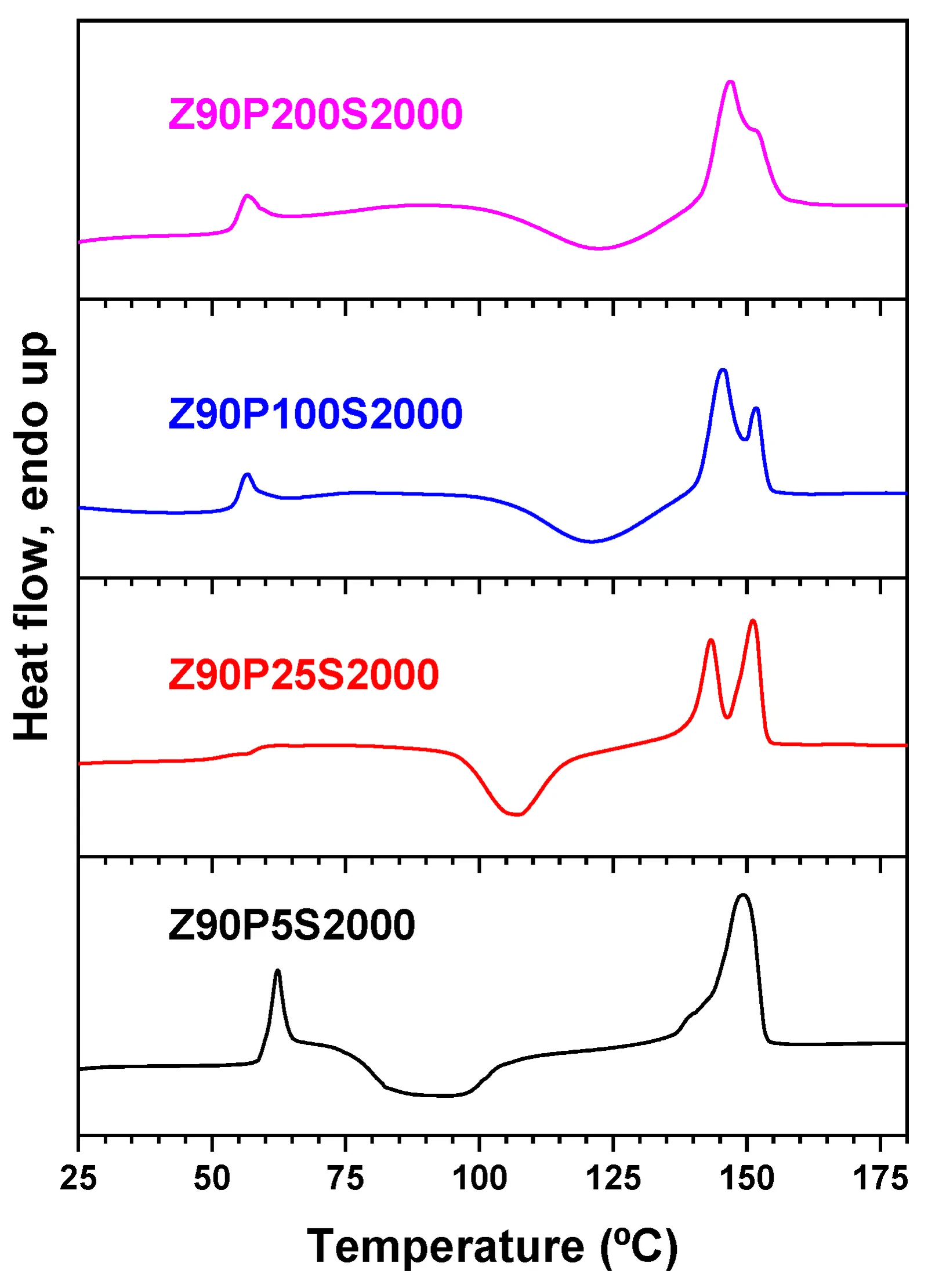
Thermograms corresponding to PLA fibers produced at different pressures (nozzle height of 200 mm, collector speed of 2000 rpm).

**Figure 12:**
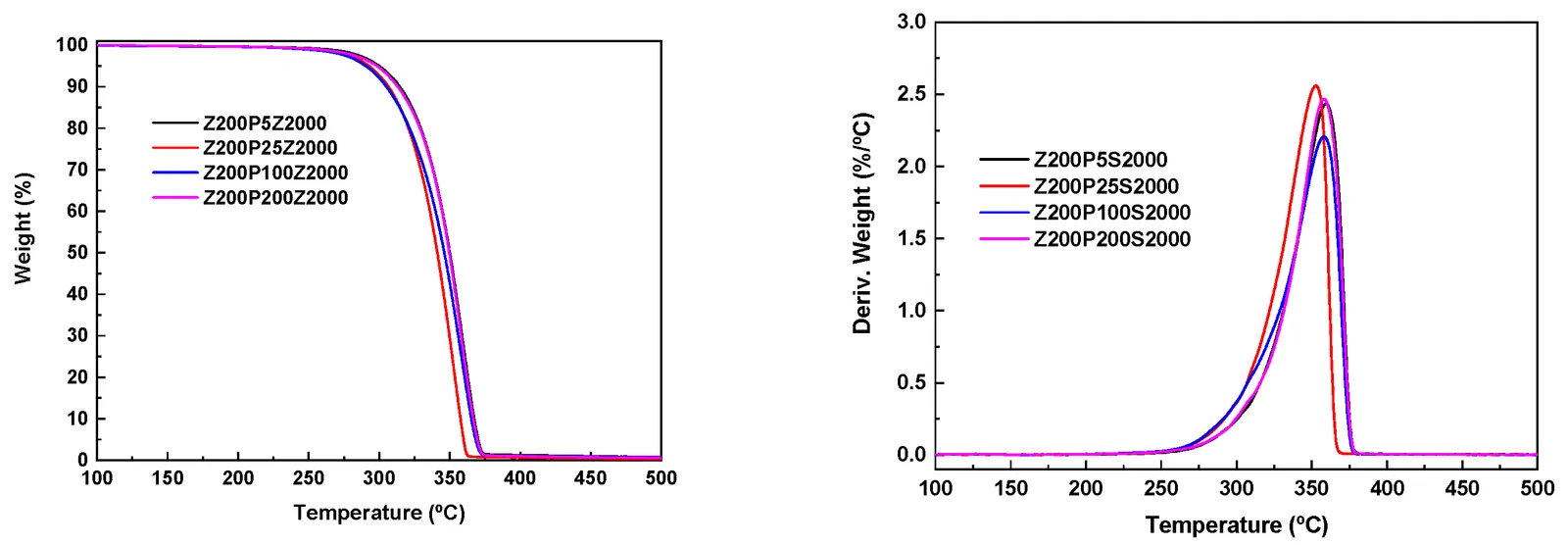
TGA curves of PLA fibers obtained at different pressures: 5 kPa, Z200P5S2000; 25 kPa, Z200P25S2000; 100 kPa, Z200P100S2000; and 200 kPa, Z200P200S2000 (nozzle height of 200 mm, collector speed of 2000 rpm).

**Figure 13:**
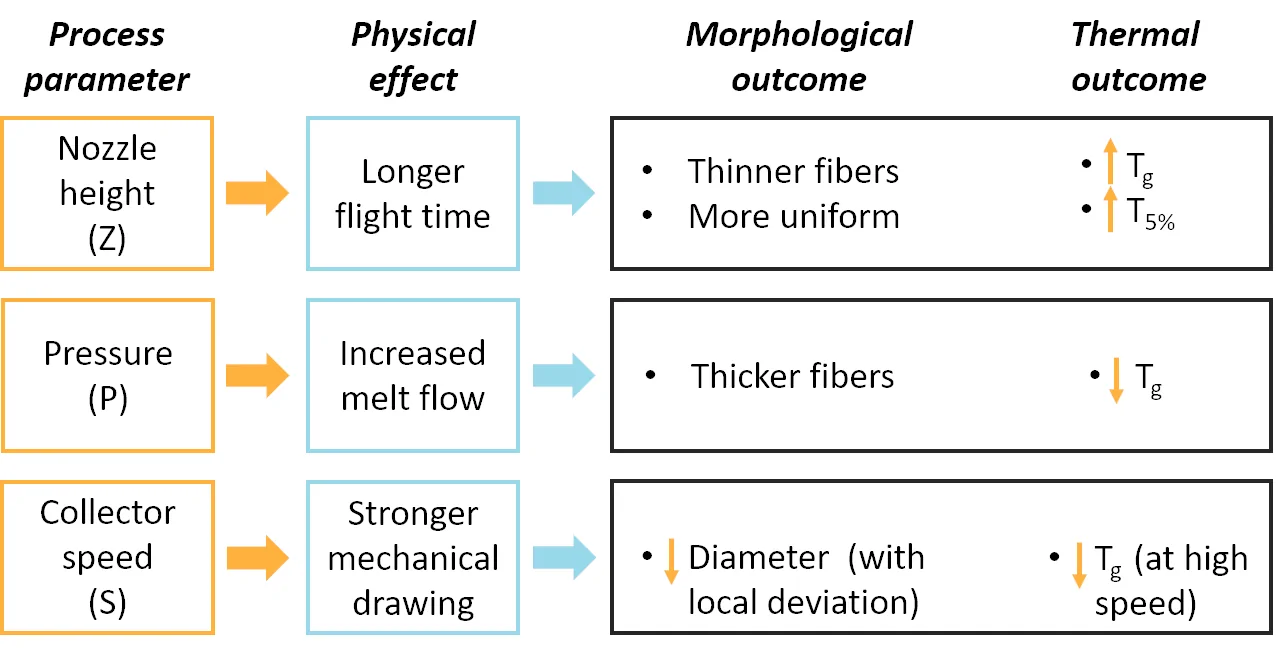
Parameter–effect map summarizing how nozzle height (Z), applied pressure (P), and collector speed (S) govern the dominant physical mechanisms during MES (flight time, melt flow, mechanical drawing). These mechanisms determine fiber diameter, uniformity, glass transition temperature (T_g_), and thermal stability (T_5%_).

**Scheme 1:**
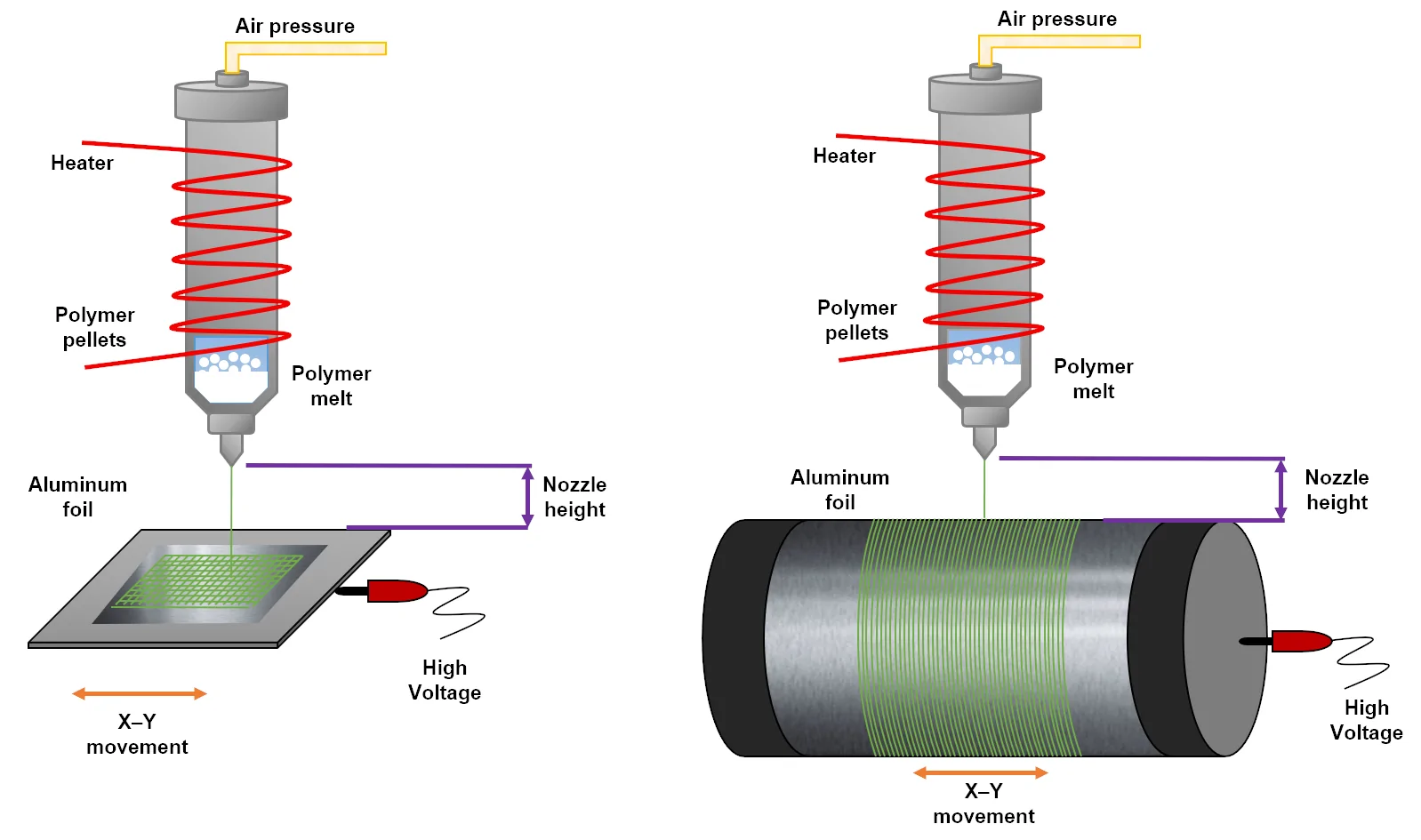
Representation of the melt electrospinning (MES)/electrowriting (MEW) process with flat and drum collectors.

**Table 1:** Thermal properties obtained from the first DSC heating run of PLA fibers: produced at different collector speeds, a constant pressure of 5 kPa, and a constant nozzle height of 90 mm: glass transition temperature (T_g_); cold crystallization temperature (T_c_^cold^); enthalpy involved in cold crystallization (∆H_c_^cold^); melting temperature (T_m_); enthalpy involved in the melting process (∆H_m_); and total crystallinity (X_c_^TOT^) developed during the whole first heating run.

Sample	T_g_ (°C)	T_c_^cold^ (°C)	ΔH_c_^cold^ (J/g)	T_m_ (°C)	ΔH_m_ (J/g)	X_c_^TOT^ (%)
Z90P5S700	54.5	117.0	26.5	142.5/149.0	26.5	0
Z90P5S1000	53.0	115.5	27.0	140.0/148.0	27.0	0
Z90P5S1500	52.5	109.5	30.5	137.5/146.5	30.5	0
Z90P5S2000	51.5	111.5	29.0	137.0/145.5	29.0	0

The transition temperatures have an error of ±0.5 °C.

**Table 2:** TGA results of PLA fibers produced at different collector speeds, a constant pressure of 5 kPa, and a constant nozzle height of 90 mm.

Sample	T_5%_ (°C)	T_10%_ (°C)	T_25%_ (°C)	T_50%_ (°C)	T_75%_ (°C)	T_MAX_ (°C)
Z90P5S700	286.5	303.5	325.0	336.5	344.0	342.5
Z90P5S1000	254.5	274.0	301.0	325.0	340.5	344.5
Z90P5S1500	262.0	284.5	310.5	335.0	350.0	353.0
Z90P5S2000	276.5	294.5	314.0	330.5	342.0	342.0

The degradation temperatures have an error of ±0.5 °C.

**Table 3:** Thermal properties of PLA fibers produced at different nozzle heights, a constant pressure of 5 kPa, and a collector speed of 2000 rpm, determined from the first DSC heating run: glass transition temperature (T_g_); cold crystallization temperature (T_c_^cold^); enthalpy involved in cold crystallization (∆H_c_^cold^); melting temperature (T_m_); enthalpy involved in melting process (∆H_m_); and total crystallinity (X_c_^TOT^) developed during the first heating run.

Sample	T_g_ (°C)	T_c_^cold^ (°C)	ΔH_c_^cold^ (J/g)	T_m_ (°C)	ΔH_m_ (J/g)	X_c_^TOT^ (%)
Z90P5S2000	51.5	111.5	25.9	137.0/145.5	25.9	0
Z120P5S2000	59.5	106.5	33.7	143.5/151.0	33.7	0
Z150P5S2000	58.5	105.5	34.2	140.5/149.5	34.2	0
Z170P5S2000	59.0	99.5	34.8	144.0/150.5	34.8	0
Z200P5S2000	60.0	87.5	37.8	139.5/149.0	37.8	0
Z220P5S2000	59.5	99.0	34.5	141.0/148.0	34.5	0

The transition temperatures have an error of ±0.5 °C.

**Table 4:** TGA results of PLA fibers produced at different nozzle heights, with a constant pressure of 5 kPa and a collector speed of 2000 rpm.

Sample	T_5%_ (°C)	T_10%_ (°C)	T_25%_ (°C)	T_50%_ (°C)	T_75%_ (°C)	T_MAX_ (°C)
Z90P5S2000	276.5	294.5	314.0	330.5	342.0	342.0
Z120P5S2000	289.0	303.0	324.0	341.5	353.0	354.0
Z150P5S2000	302.0	316.5	335.5	351.0	362.0	360.5
Z170P5S2000	304.0	317.5	332.5	346.0	356.0	355.5
Z200P5S2000	300.0	314.5	334.5	350.0	361.0	359.5
Z220P5S2000	294.5	308.5	328.0	342.5	352.5	351.5

The degradation temperatures have an error of ±0.5 °C.

**Table 5:** Thermal characteristics of fibers obtained at different pressures (nozzle height of 200 mm, collector speed of 2000 rpm), deduced from the first DSC heating run: glass transition temperature (T_g_); cold crystallization temperature (T_c_^cold^); enthalpy involved in cold crystallization (∆H_c_^cold^); melting temperature (T_m_); enthalpy involved in melting process (∆H_m_); and total crystallinity (X_c_^TOT^) developed during the first heating run.

Sample	T_g_ (°C)	T_c_^cold^ (°C)	ΔH_c_^cold^ (J/g)	T_m_ (°C)	ΔH_m_ (J/g)	X_c_^TOT^ (%)
Z200P5S2000	60.0	87.5	33.9	139.5/149.0	33.9	0
Z200P25S2000	58.0	107.5	25.4	143.0/151.0	25.4	0
Z200P100S2000	54.5	121.0	24.1	145.5/151.5	24.1	0
Z200P200S2000	55.0	122.5	22.0	147.0/152.0	22.0	0

The transition temperatures have an error of ±0.5 °C.

**Table 6:** TGA results of PLA fibers produced at different pressures (nozzle height of 200 mm, collector speed of 2000 rpm).

Sample	T_5%_ (°C)	T_10%_ (°C)	T_25%_ (°C)	T_50%_ (°C)	T_75%_ (°C)	T_MAX_ (°C)
Z200P5S2000	300.0	314.5	334.5	350.0	361.0	359.5
Z200P25S2000	292.5	306.5	325.5	341.0	352.0	353.0
Z200P100S2000	290.5	305.0	327.0	346.0	358.0	358.5
Z200P200S2000	298.5	313.5	334.0	349.5	360.0	358.0

The degradation temperatures have an error of ±0.5 °C.

## Data Availability

The raw data supporting the conclusions of this article will be made available by the authors upon request.
